# A Practical Treatise on Diseases of the Skin

**Published:** 1902-01

**Authors:** 


A Practical Treatise on Diseases of the Skin. By John V. Shoemaker, M. D., LL. D., Professor of Skin and Venereal Diseases in the Medico-Chirurgical College and Hospital of Philadelphia, etc. Fourth edition, revised and enlarged, with chromogravure plates and other illustrations. Pages, 892; price, $5.00. New York: D. Appleton & Co. 1901.

This edition embraces as new features the advances made in dermatology during the last three years. Every source of information has been drawn upon and the most careful revision has been made of the entire work. Improved methods of treatment have been incorporated as well as new points of symptomatology. The work is well established and is entitled to be called a standard. A larger list of remedies follows the discussion of each disease than appears in any other work with which the reviewer is acquainted.

